# Influence of a 6-month physical training program on serum and urinary concentrations of trace metals in middle distance elite runners

**DOI:** 10.1186/s12970-019-0322-7

**Published:** 2019-11-14

**Authors:** M. Maynar, I. Bartolomé, J. Alves, G. Barrientos, F. J. Grijota, M. C. Robles, D. Muñoz

**Affiliations:** 1 Sport Sciences Faculty, University of Extremadura, Avenida de la Universidad s/n 10003, Cáceres, Spain; 2 Education Faculty, University of Salamanca, / Henry Collet, 52-70, 37007 Salamanca, Spain; 3 Education Faculty, University of Extremadura, Avenida de la Universidad s/n 10003, Cáceres, Spain

**Keywords:** Minerals, Essential trace elements, Blood, Urine, Exercise, Training

## Abstract

**Background:**

The aim of this survey was to determine the effects of an aerobic physical training program of six months duration on the serum and urinary concentrations of essential trace elements among middle distance runners and untrained, non-sportsmen participants.

**Methods:**

24 well-trained, middle-distance (1500 and 5000 m), aerobic male runners (AG) were recruited at the beginning of their training season and 26 untrained males formed the control group (CG). All participants were from the same region of Spain, and all of them had been living in this area for at least two years. Serum and urine of samples of Cobalt (Co), Copper (Cu), Manganese (Mn), Molybdenum (Mo), Selenium (Se), Vanadium (V) and Zinc (Zn) were obtained at the beginning of the training season, and six months later, from all participants. All samples were analyzed with inductively coupled plasma mass spectrometry (ICP-MS).

**Results:**

Two-way ANOVA showed significant differences relative to group effect in serum concentrations of Co, Cu, Mn, Mo, Se and Zn. Attending to time effect, there were differences in Mn (*p* = 0.003) and Zn (*p* = 0.001). The group x time interaction revealed differences only in the case of Mn (*p* = 0.04). In urine, significant differences between group were obtained in Co, Cu, Mn, Se and V. Time effect showed changes in Co, Cy, Mo and Se. Finally, the group and time interaction revealed significant differences in urinary Cu (p = 0.001), Mn (*p* = 0.01) and Se (p = 0.001).

**Conclusions:**

A six-month aerobic training program for well-trained athletes induced modifications in the body values of several minerals, a fact which may reflect adaptive responses to physical exercise. The obtained data could be interesting for physicians or coaches in order to consider specific modifications in sportsmen’s diets as well as to determine specific nutritional supplementation strategies.

## Introduction

The essential trace metals are necessary for a wide range of body functions, developing key roles in the adaptation to exercise as well as to the normal physiological behavior of the body.

In this respect, cobalt (Co) is an essential element, present in the composition of vitamin B_12_ that enhances erythropoiesis [1]. Furthermore, Co dilates the vessels and has a hypotensive effect [2].

Copper (Cu) is essential in the composition of the mitochondrial cytochrome-*c* oxidase, an enzyme which catalyzes the final step in aerobic respiration [3]. In addition, three Cu enzymes (ceruloplasmin, cytosolic superoxide dismutase (SOD), and extracellular SOD) develop important antioxidant functions [4, 5]. Mitochondrial SOD, a manganese (Mn) containing enzyme, protects the mitochondria against the action of free radicals [6].

The enzyme xanthine oxidase depends on molybdenum (Mo), is critical in the production of uric acid, and is considered another important cellular antioxidant.

Glutathione peroxidase (GPx) is a selenium (Se) dependent enzyme, and acts to protect cells against hydrogen peroxide [7].

One of the most best known biological effects of vanadium (V) is its insulin-mimetic properties that occur in the majority of intact cellular systems [8].

Regarding zinc (Zn), cytosolic SOD is a zinc (Zn) dependent enzyme that protects cells from the superoxide anion and develops important exercise-induced adaptations, like the protection of the mitochondria or other subcellular organelles [9].

Recently, it has been found that physical training can induce adaptive responses, which may be reflected in the body values of some essential trace elements. These responses seem to depend on the modality of exercise practiced (aerobic, aerobic-anaerobic or anaerobic) [10–13]. The authors observed a higher basal concentration in serum of Mo, Cu, Mn and Zn, and lower in Co and Se in athletes than controls.

In all cases, current information about the long-term effect of continuous physical training on the serum or urinary concentrations of essential trace elements is limited and more research is required in this field.

Thus, the aim of the present study was to determine if athletes present different concentrations of essential minerals (Co, Cu, Mn, Mo, Se, V and Zn) with respect to sedentary people and if there are exercise-induced modifications in the serum and urinary concentrations as a result of a period of six months of intense, predominantly aerobic, physical training.

## Materials and methods

### Participants

Twenty-six Spanish national medium-distance runners (AG) (21 ± 4 years) were recruited at the start of their training period. All of them had been competing in 1500 and 5000 m race modalities.

The athletes had been performing aerobic physical training regularly for the previous two years, developing an average volume of 120 km per week of rigorous training aimed at high-level competition. Their weekly training routines consisted of 3–4 days of aerobic continuous running and 2–3 days of aerobic-anaerobic fartlek or intense series.

Of the twenty-six athletes that began the study, two athletes dropped out due to sports injuries produced during the training period. The control group (CG) consisted of twenty-six untrained, male non-sportsmen (21 ± 3 years) who only had been leading a normal, active lifestyle. Their physical activities consisted of recreational football, handball or basketball, recording a weekly volume of less than 2 h. The anthropometric characteristics of both groups are described in Table 1.

**Table 1:** Nutritional intake of elements in controls and athletes at baseline and after the training program

		Time		ANOVA (p)		
Nutritional intake		Baseline	6 months	Group effect	Time effect	*Group x Time interaction*
Water (g)	CG (*n* = 26)	1010.12 ± 300.22	1054.33 ± 320.05	0.15	0.12	0.38
	AG (*n* = 24)	1044.49 ± 288.31	1037.15 ± 334.41			
Energy (Kcal)	CG (*n* = 26)	2847 ± 930	2844 ± 1000	0.21	0.34	0.51
	AG (*n* = 24)	2884 ± 914	2535 ± 400*			
Proteins (g)	CG (*n* = 26)	127.5 ± 43.3	128.1 ± 39.9	0.43	0.45	0.88
	AG (*n* = 24)	129.7 ± 41.1	108.4 ± 17.6			
Lipids (g)	CG (*n* = 26)	81.15 ± 21.4	85.73 ± 30.50	0.12	0.18	0.30
	AG (*n* = 24)	90.07 ± 32.31	87.62 ± 36.91			
Carbohydrates (g)	CG (*n* = 26)	360.7 ± 75.43	359.80 ± 72.07	0.34	0.22	0.45
	AG (*n* = 24)	357.5 ± 71.39	332.4 ± 78.9			
Co (0.2–0.3 mg/d)	CG (*n* = 26)	0.25 ± 0.13	0.26 ± 0.16	0.32	0.19	0.55
	AG (*n* = 24)	0.22 ± 0.14	0.27 ± 0.19			
Cu (1–3 mg/d)	CG (*n* = 26)	1.84 ± 0.63	1.81 ± 0.70	0.27	0.72	0.62
	AG (*n* = 24)	1.72 ± 0.61	1.60 ± 0.56			
Mn (2.5–5 mg/d)	CG (*n* = 26)	3.04 ± 1.31	3.31 ± 1.97	0.32	0.65	0.87
	AG (*n* = 24)	3.23 ± 1.41	3.27 ± 1.60			
Mo (0.07–0.4 mg/d)	CG (*n* = 26)	0.29 ± 0.14	0.27 ± 0.18	0.55	0.34	0.78
	AG (*n* = 24)	0.27 ± 0.17	0.33 ± 0.21			
Se (0.05–0.2 mg/d)	CG (*n* = 26)	0.11 ± 0.018	0.09 ± 0.017	0.35	0.47	0.65
	AG (*n* = 24)	0.096 ± 0.018	0.089 ± 0.086			
V (10–70 μg/d)	CG (*n* = 26)	15.66 ± 18.21	15.98 ± 17.62	0.41	0.30	0.61
	AG (*n* = 24)	13.44 ± 17.19	31.29 ± 37.60			
Zn (10–15 mg/d)	CG (*n* = 26)	14.21 ± 5.01	13.89 ± 5.48	0.10	0.07	0.15
	AG (*n* = 24)	11.76 ± 5.94	10.18 ± 2.32			

*CG* Control group, *AG* Athletes group. Values are presented as mean ± standard deviation

During the six months of the training period the athletes ran a total of approximately 3537.85 km in training and competitions, varying the intensities from moderate (aerobic threshold) to high (anaerobic threshold or higher). The training was configured with 3–4 days of continuous running or fartlek and 2–3 days of more intense series, depending on if there was a competition over the weekend. Low intensity, regenerative exercise was performed the day after a competition. The control group continued with their normal daily activities during the whole experimental period. None of the controls followed any specific physical training program.

A GPS pack equipped with pulsometers (Polar. Norway) was used to track the training loads during the survey. The GPS were lent to the sportsmen at the beginning of the survey and the researchers recorded and analyzed their training routines every week.

All the participants had been living in the same geographic area of Spain for at least two years. The present study was approved by the bioethics committee of the University of Extremadura under the Helsinki Declaration ethic guidelines of 1975, updated at the World Medical Assembly in Seoul 2008, for investigations involving human subjects. All the participants were explained the purpose of the study and gave their informed consent.

### Anthropometric measurement

The morphological characteristics of the participants were measured in the morning and always at the same time and under identical conditions. Body height was measured to the nearest 0.1 cm using a wall-mounted stadiometer (Seca 220. Hamburg. Germany). Body weight was measured to the nearest 0.01 kg using calibrated electronic digital scales (Seca 769. Hamburg. Germany) in nude, barefoot conditions. Body fat content was estimated from the sum of 6 skinfolds (∑6) (abdominal, suprailiac, tricipital, subscapular, thigh and calf skinfolds). Skinfold thicknesses were measured with a Harpenden caliper (Holtain Skinfold Caliper. Crosswell, UK). All measurements were made by the same operator, skilled in kinanthropometric techniques, in accordance with the International Society for the Advancement of Kinanthropometry recommendations. Heart rate and blood pressure were determined using an automatic sphygmomanometer (Omron HEM-780. Osaka. Japan) by a skilled technician, always after a five-minute rest period in a supine position.

### Nutritional evaluation

All participants completed a dietary questionnaire in order to ensure that they were not taking any vitamins, minerals or other supplements and in order to guarantee that they were following a similar diet. The questionnaire consisted of a 3-day, daily nutritional record, filled out on two pre-assigned weekdays and on one weekend day.

On each day, all participants recorded the amount (in grams) of each food consumed in every meal ingested on every one of the three days. Once completed, every questionnaire compiled the total amount of each food consumed, grouped by meals. Then the nutritional composition of their diets was evaluated using different food composition tables [14–16]. These tables contain nutritional information about all kinds of foods. The nutritional questionnaires were applied at the start and at the end of the study period.

None of the participants followed a specific diet, nutritional plan or specific supplementation during the whole survey.

### Physical performance test

An exercise test was used to evaluate the performance variables for each participant. The test consisted of running on a treadmill (Powerjoc. UK) until voluntary exhaustion. The ergospirometric and cardiovascular variables were measured using a gas analyzer (Metamax. Cortex Biophysik. Gmbh. Germany) and a Polar pulsometer (Polar. Norway). To guarantee a warm-up phase before the test, all participants ran progressively for 15 min, ending at the initial speed of the test. Then, the participants performed the exercise test. Control participants performed 5 min at 6 km/h, 5 min at 7 km/h and 5 min at 8 km/h to ensure a proper warm-up phase. Athletes ran at 8, 9 and 10 km/h respectively. The participants then performed the exercise test. The protocol consisted in running incrementally in stages, until voluntary exhaustion (no possibility of continuing to run) starting at an initial speed of 8 km/h for controls and 10 km/h for athletes and increasing the speed by 1 km/h every 400 m, with a stable slope of 1%. The anaerobic threshold was determined using the ventilatory parameters method. This test was used to run a sufficient distance in order to achieve the same physiological changes which should be expected to occur in a field test. All tests were performed in the morning (between 10 and 12 a.m.) within the recommended parameters [17]. Training intensity and volume were reduced the two previous days applying a regenerative load in order to avoid fatigue in the physical tests.

The exercise test was performed at the beginning and at the end of the experimental period, with the time and conditions being the same for each participant.

### Sample collection

At nine o’clock in the morning 5 mL of venous blood were drawn from each participant using a plastic syringe fitted with a stainless-steel needle. The blood samples were collected in a metal-free polypropylene tube (previously washed with diluted nitric acid). Then, the blood samples were centrifuged at 3000 rpm for 15 min at room temperature to separate the serum. Once isolated, the serum was aliquoted into an Eppendorf tube (previously washed with diluted nitric acid) and was conserved at − 80 °C until further analysis. Morning midstream urine samples were obtained from all subjects and were collected in polyethylene tubes previously washed with diluted nitric acid and frozen at − 80 °C until analysis. Prior to analysis, the samples were thawed and homogenized by shaking. This protocol was applied at the beginning and at the end of the experimental period.

### Experimental design

#### Urinary creatinine determination

Creatinine concentrations were measured in all urine samples to determine different dilution degrees [18], using Sigma’s Creatinine 555–A kit and a UNICAM 5625 spectrophotometer.

#### Serum and urinary trace element determination

##### Sample preparation

Co, Cu, Mn, Mo, Se, V and Zn analyses were performed by inductively coupled plasma mass spectrometry (ICP-MS). To prepare the analysis, the organic matrix was decomposed by heating it for 10 h at 90 °C after the addition of 0.8 mL HNO_3_ and 0.4 mL H_2_O_2_ to 2 mL of serum or urine samples. The samples were then dried at 200 °C on a hot plate. Samples reconstitution was carried out by adding 0.5 mL of nitric acid, 10 μL of Indium (In) (10 mg/L) as the internal standard, and ultrapure water to complete 10 mL.

##### Standard and reference material preparation

Reagent blanks, element standards and certified reference material (Seronorm, lot 0511545, AS Billingstand, Norway) were prepared identically, and used for accuracy testing. Before the analysis, the commercial control materials were diluted according to the recommendation of the manufacturer.

##### Sample analysis

Digested solutions were assayed with an ICP-MS Nexion model 300D (PerkinElmer, Inc., Shelton, CT, USA) equipped with a triple quadrupole mass detector and a reaction cell/collision device that allows operation in three modes: without reaction gas (STD); by kinetic energy discrimination (KED) with helium as the collision gas; and in reaction mode (DRC) with ammonia as the reaction gas. Both collision and reaction gases such as plasmatic argon had a purity of 99.999% and were supplied by Praxair (Madrid, Spain). Two mass flow controllers regulated gas flows. The frequency of the generator was free-swinging and worked at 40 Mhz. Three replicates were analyzed per sample. The sample quantifications were performed with indium (In) as the internal standard. The values of the standard materials of each element (10 μg/L) used for quality controls were in agreement with intra and inter-assay variation coefficients of less than 5%.

#### Statistical evaluations

Statistical analyses were carried out with IBM SPSS Statistics 22.0 for Windows. The results are expressed as means ± standard deviations. Normality was tested by Shapiro– Wilk test. Two-way ANOVA was used to show differences between study variables. The level of significance was set at *p* < 0.05.

## Results

### Dietary habits

Table 1 shows the results of the nutritional evaluations. None of the participants followed any special diet like e.g., vegetarians and vegans. None of them consumed any mineral supplements either. They reported a similar intake of milk, fish, meat, fruits, and vegetables during the training period. As can be observed in Table 1, no differences were found between groups in any of the nutritional variables at baseline, but the caloric content of the diet was lower at the end of the training period in AG group than CG. In this sense, when examining the time effect, no differences were observed.

### Anthropometric and ergospirometric characteristics of participants

Table 2 shows the anthropometric and cardiorespiratory variables of CG and AG at baseline and after the experimental period. Significant differences were observed between groups, being higher the values of total weight (*p* = 0.001), ∑6 skinfolds (p = 0.001) in CG with respect to AG. Also, AG presented higher values of VO_2_ max (*p* = 0.001) and VE max (p = 0.001), and lower values of Rest HR (p = 0.001) than CG. Attending to time effect, no differences were observed after the training period. By interaction effect, we mean the combined effects of factors on the dependent variable. As Table 2 shows, this interaction effect was not significative.

**Table 2:** Ergoespirometrics results of controls and athletes at baseline and after the training program

		Time		ANOVA (p)		
Parameters		Baseline	6 months	Group effect	Time effect	*Group x Time interaction*
Total Weight (Kg)	CG (*n* = 26)	76.94 ± 11.07	77.62 ± 12.14	0.001	0.2	0.11
	AG (*n* = 24)	65.55 ± 7.55	64.73 ± 7.83			
∑6 Skinfold (mm)	CG (*n* = 26)	62.76 ± 12.23	63.02 ± 13.05	0.001	0.08	0.13
	AG (*n* = 24)	48.74 ± 11.17	46.59 ± 8.89			
Rest HR (b/min)	CG (*n* = 26)	65.12 ± 12.42	67.34 ± 11.71	0.001	0.13	0.21
	AG (*n* = 24)	52.12 ± 12.42	49.87 ± 9.27			
HR max (b/min)	CG (*n* = 26)	196.33 ± 7.55	197.41 ± 8.01	0.42	0.51	0.72
	AG (*n* = 24)	193.69 ± 7.85	194.30 ± 7.50			
VO_2_ max (ml/kg/min)	CG (*n* = 26)	45.72 ± 7.51	46.32 ± 8.16	0.001	0.2	0.31
	AG (*n* = 24)	66.46 ± 10.12	67.94 ± 8.10			
VE max (L/min)	CG (*n* = 26)	98.66 ± 11.47	99.26 ± 16.63	0.001	0.42	0.25
	AG (*n* = 24)	137.95 ± 55.34	129.00 ± 26.92			

*CG* Control group, *AG* Athletes group. *HR* Hearth rate, *VO*_*2*_ Oxygen uptake, *VE* Pulmonary ventilation

Values are presented as mean ± standard deviation

### Serum concentrations of metals

Table 3 shows the serum concentrations of each metal at the start and end of the study in both groups. The ANOVA showed significant differences in Co, Cu, Mn, Mo, Se and V between groups. In addition, there were significant differences in Mn (*p* = 0.003) an Zn (*p* = 0.001) across time. We observed a time x group interaction for serum concentration of Mn (*p* = 0.04). Thus, a decrease in this mineral was provoked in AG by training period.

**Table 3:** Serum concentrations of trace elements in controls and athletes at baseline and after the training program

		Time		ANOVA (p)		
Minerals		Baseline	6 months	Group effect	Time effect	*Group x Time interaction*
Co (μg/L)	CG (*n* = 26)	0.81 ± 0.40	0.83 ± 0.51	0.001	0.52	0.61
	AG (*n* = 24)	0.68 ± 0.10	0.67 ± 0.11			
Cu (μg/L)	CG (*n* = 26)	750.5 ± 121.5	760.4 ± 113.8	0.01	0.33	0.3
	AG (*n* = 24)	721.5 ± 119.5	675.4 ± 152.2			
Mn (μg/L)	CG (*n* = 26)	1.84 ± 0.41	1.98 ± 1.36	0.01	0.003	0.04
	AG (*n* = 24)	3.48 ± 1.50	1.36 ± 1.00			
Mo (μg/L)	CG (*n* = 26)	0.33 ± 0.15	0.34 ± 0.12	0.01	0.2	0.21
	AG (*n* = 24)	0.66 ± 0.72	0.51 ± 0.27			
Se (μg/L)	CG (*n* = 26)	119.13 ± 14.37	118.43 ± 13.37	0.001	0.22	0.14
	AG (*n* = 24)	98.43 ± 13.37	92.30 ± 13.74			
V (μg/L)	CG (*n* = 26)	0.54 ± 0.61	0.53 ± 0.71	0.2	0.51	0.52
	AG (*n* = 24)	0.56 ± 0.50	0.10 ± 0.13			
Zn (μg/L)	CG (*n* = 26)	979.6 ± 114.41	968.3 ± 116.61	0.05	0.001	0.1
	AG (*n* = 24)	658.4 ± 92.61	859.3 ± 125.3			

*CG* Control group, *AG* Athletes group. Values are presented as mean ± standard deviation

### Urinary concentrations of metals

Table 4 shows the urinary concentrations of each metal at the start and at the end of the training period in both study groups. When examining the group effect, there were significant differences in the urinary excretion of all minerals except in the case of Mo. However, after the training period, significant differences were obtained in Cu (*p* = 0.001), Mn (*p* = 0.01), and Se (p = 0.001).

**Table 4:** Urinary concentrations of elements in controls and athletes at baseline and after the training program

		Time		ANOVA (p)		
Minerals		Baseline	6 months	Group effect	Time effect	*Group x Time interaction*
Co (μg/g)	CG (*n* = 26)	0.43 ± 0.66	0.44 ± 0.70	0.001	0.07	0.09
	AG (*n* = 24)	0.09 ± 0.08	0.33 ± 0.30			
Cu (μg/g)	CG (*n* = 26)	19.66 ± 16.8	20.5 ± 14.5	0.001	0.001	0.001
	AG (*n* = 24)	82.3 ± 47.2	38.19 ± 16.12			
Mn (μg/g)	CG (*n* = 26)	5.48 ± 3.59	4.99 ± 3.91	0.002	0.01	0.01
	AG (*n* = 24)	1.33 ± 3.20	12.44 ± 16.98			
Mo (μg/g)	CG (*n* = 26)	34.42 ± 29.71	34.66 ± 30.72	0.51	0.12	0.13
	AG (*n* = 24)	41.82 ± 16.64	38.07 ± 25.51			
Se (μg/g)	CG (*n* = 26)	23.49 ± 13.54	22.98 ± 13.37	0.001	0.001	0.001
	AG (*n* = 24)	22.13 ± 6.09	18.99 ± 7.200			
V (μg/g)	CG (*n* = 26)	1.06 ± 0.78	0.96 ± 0.68	0.001	0.6	0.33
	AG (*n* = 24)	0.93 ± 0.30	1.08 ± 1.47			
Zn (μg/g)	CG (*n* = 26)	261.4 ± 214.61	258.2 ± 207.74	0.28	0.07	0.08
	AG (*n* = 24)	134.4 ± 110.1	264.75 ± 200.85			

*CG* Control group, *AG* Athletes group. Values are presented as mean ± standard deviation

Finally, a time x group interaction was observed in urinary excretion of Cu (0.001), Mn (p = 0.01) and Se (p = 0.001), decreasing the urinary excretion of Cu and Se in AG, and increasing in the case of Mn.

## Discussion

This study aimed to determine if athletes present different concentrations of essential minerals (Co, Cu, Mn, Mo, Se, V and Zn) with respect to sedentary people and if exercise-induced modifications in the serum and urinary concentrations as a result of a period of six months of intense, predominantly aerobic, physical training.

Thus, the discussion of the results related to the mineral elements studied will be presented, as in the results, analyzing the possible differences between both groups, and then the effects that the 6 months of the study caused in both groups.

All participants lived in the same region and were the same age, this helped to avoid several factors which could have influenced the results. In this respect, control participants did not suffer any anthropometric or ergospirometric change, a fact which reinforces the previous statement.

The information used to evaluate the diets and ascertain the specific amounts consumed by the participants is a critical point in this kind of studies. The present survey used different food composition tables [14–16]. In order to ensure reliability, and considering the high variability of amounts of minerals per food reported in the literature, the average amount of minerals in each food was calculated using the information in the literature.

When basal results were analyzed, both groups presented a similar intake of nutrients at the start of the study (Table 1), but the caloric intake was lower at the end of the training period in the AG than CG. Table 2 shows, as expected, that in high-level athletes (AG), weight, body fat and resting heart rate were significantly lower compared to the CG and, on the contrary, the ergospirometric parameters VO_2_ max and VE max were much higher in the AG athletes regarding the CG, staying similar at the end of the study. These differences are due to the adaptations that aerobic training produces in athletes.

In relation to the elements analyzed, Tables 3 and 4 show that all serum and urinary metal concentrations were within the normal values reported in previous surveys [10, 12], developed with a similar technique and expressed in the same units (μg/L).

Serum Co concentration was similar in both groups, but urinary concentration was significantly higher in CG than AG. These results are similar to those found by Muñoz et al. (2019), also in high-level athletes, indicating that they could be due to an adaptive process to maintain normal values in serum and avoid a deficit of the element that could have negative consequences for the maintenance of erythropoiesis [12].

Cu is an essential element in the structure of the important enzyme Cu-Zn-SOD. This enzyme protects the athlete against superoxide anion and is commonly synthesized in large amounts among aerobic sportsmen, suggesting a specific exercise-induced metabolic adaptation [9]. Our results show higher values in urine of this element in AG than CG at baseline and the end of the study. However, the main effect was observed between groups. Three previous studies indicated that physical exercise results in large increases in urinary excretion of Cu [12, 19, 20]. As shown by Muñoz et al. (2019), the increased urinary Cu obtained in this survey among AG participants may be related to the biological mobilization of this mineral induced by physical training, as has been described previously [20].

It has been reported that physical exercise increases the activity of Mn-SOD at the myocardial level. So, it has been suggested that the exercise increases the activity of Mn-SOD and that it could be linked to a diminution in the serum concentrations of Mn [21–23].

Furthermore, Mn is an integral part of other important metabolic enzymes such as pyruvate carboxylase, a key enzyme in the process of gluconeogenesis [24]. This enzyme acts by regulating the whole activity of the Krebs Cycle, using acetyl-CoA as an allosteric activator. Mn is also an integral component of arginase. This enzyme requires two molecules of Mn to develop an appropriate function. It takes part in the metabolism of urea, converting L-arginine into L-ornithine, and L-ornithine into urea [25, 26]. The metabolism of urea is a critical point in endurance exercise, as this chemical compound is an end product in protein metabolism. In this respect, it has been reported that endurance exercise may lead to an increased protein catabolism and affect endurance performance, muscle strength and physical fitness [25].

The high serum Mn concentration found at the start of the study, but not at the end, in AG participants, is similar to other studies and could also be caused by a possible iron deficiency in athletes [10, 12, 13], a fact that would increase Mn absorption, as indicated by Park et al. (2013) [27] or a decrease in urinary elimination in AG. However, it is interesting to highlight that at the end of the study the serum concentration of Mn in the AG is similar to those of the CG, accompanied by a significant increase in urinary elimination, which would reveal a possible renal adaptation with training. This response could be produced in order to keep a normal serum concentration of this element.

Mo participates in oxide-reduction processes as an integral part of several enzymes like xanthine dehydrogenase, an enzyme which catalyzes the hypoxanthine transformation of xanthine to uric acid which is considered an antioxidant [28, 29]. Our results show higher serum concentrations in AG at baseline and final of the training period, with no changes in urinary concentrations.

In a previous study, Maynar et al. (2018) found significantly elevated values of Mo in all the sports modalities studied with respect to the control group being the lowest in the aerobic athletes. For them, the augmented Mo concentrations would ease the formation of uric acid as well as decrease the damage caused by superoxide anions generated by xanthine oxidase in ischemia-reperfusion processes, a situation induced by high intensity muscular activities [11, 30].

Se is an essential element which takes part in several biochemical processes of the antioxidant metabolism. In relation to the effect of exercise on the antioxidant system, previous studies have concluded that physical training improves the antioxidant response, a fact which has been reported to be reflected in a reduced lipid peroxidation among trained athletes throughout the season [30]. Furthermore, Se is an integral component in the catalytic space of the enzyme GPx, so changes in their serum concentrations may influence the activity of this enzyme [31, 32], by mean of a reduced bioavailability of this mineral. This enzyme also develops an important role in protecting against oxidative stress and lipid peroxidation as well, and it is also responsible for the detoxification of lipid peroxides and hydrogen peroxide (H_2_O_2_) [32–34]. In this respect, an increase in the amounts of this enzyme in the erythrocyte has been reported as a response to high-level physical training [9], which may affect the metabolism of Se.

In our study, serum concentrations of Se were significantly lower in AG that CG at baseline and final of the training period, with a similar urinary excretion at the start. However, we found a significant decrease in urinary elimination in AG than CG at the end of the study. The same results were obtained by Maynar et al. (2018) and Sánchez et al. (2010) who found lower Se values in an active population in comparison to sedentary people [10, 35]. It could be that Se intake from food was not enough to maintain the constant levels of blood Se during training [36]. The main reason for this affirmation is that Se requirements are increased among athletes [37]. The decrease in urinary elimination would be related to a possible adaptive mechanism to avoid greater losses of Se that would be harmful for the athletes.

V is also closely linked to exercise metabolism, as within its biological properties it includes an insulin-mimetic role [8, 38]. In this respect, Seale et al. (2006) reported that the effects of V on the insulin response are based on a stimulation of insulin sensitization, reinforced by a stimulation of adiponectin secretion from the adipocytes, as adiponectin is a hormone rich in V [39]. Similar serum and urinary levels of V were found in both groups in basal conditions before and after the study.

Zn is probably one of the most known trace elements in the field of exercise physiology. This element is an integral compound in the structure of more than 70 enzymes involved in several cellular functions, like the metabolism of carbohydrates (glycolysis and gluconeogenesis), lipid, proteins, and DNA. In addition, Zn may develop an antioxidant effect by itself and may help to prevent oxidative processes by means of an antagonistic role against active metals involved in oxidation-reduction reactions, such as iron and copper [40]. Furthermore, Zn also performs an important anti-inflammatory function by reducing cytokine production [41] and it has been reported that high concentrations in serum Zn are associated with a decreased production of lactate and higher blood glucose values during exercise (Khaled et al., 1997), because lactate dehydrogenase is an enzyme that contains Zn [42]. In this respect, adequate concentrations in serum Zn may facilitate the reduction of lactate to pyruvate facilitating the action of LDH activity in muscle, reducing muscle fatigue [43]. In the present survey, Zn concentrations determined in serum and urine showed significantly lower serum and urinary concentrations in the athletes.

At the beginning of the study, our athletes presented values of Zn similar to those found by Maynar et al. (2018b) in aerobic athletes and that were also significantly lower than in the respective controls, indicating that the low serum concentrations among athletes, may be due to an exercise-induced body Zn redistribution between body stores, bloodstream and tissues [11]. The urinary concentrations were similar to those presented by Maynar et al. (2018), indicating that this lower elimination could correspond to an adaptive mechanism to avoid element losses [13].

Regarding the second section of the discussion an important issue in the research with high-level athletes, because of the high training intensities, is attrition and fatigue, which may affect the results. In this sense, HR (resting and maximal) and VO_2_ max can be valid parameters to identify fatigue and overtraining [44, 45] . As can be observed in Table 2 no differences were evident among the athletes at the end of the survey, in comparison to the respective initial values. Furthermore, none of the athletes presented symptoms of overtraining.

The control group did not practice any kind of sport and their nutritional demands were stable during the whole experimental period. This fact served to verify the nutritional analysis, as well as to have a nutritional reference of a population of non-sportsmen. None of the diets of any of the participants were manipulated by the researchers.

According to the data from the diets (Table 1), CG did not experience any change while the athletes showed a diminution (*p* < 0.05) in the caloric intake at the end of the experimental period, a fact which was accompanied by an increase in the intake of V.

Regarding the body values of minerals after the six months of the study, no changes were observed among CG either in serum or urine.

When we observe the possible changes occurring in serum and urinary concentrations of minerals, an increase in urinary Co elimination was reported in athletes without modifications in the dietary intake and serum values. It could be due to an increase in the degradation of cobalamin, a Co containing vitamin, as a consequence of physical training, a fact which has not been demonstrated yet.

On the other hand, no changes were reported in the ingestion or serum values of Cu after training, so the decrease found in the urinary elimination among the athletes could be explained as a body response to retain this element and ensure adequate amounts which would allow the body to overcome the metabolic demands induced by physical training, like enzymatic production. Similarly, no changes in serum concentrations of Cu were found in other studies [46].

The diminution of Mn observed in the serum after the training period of the athletes, could mainly be due to a possible body redistribution of this element to meet cellular exercise-induced demands. This diminution in serum was accompanied by an increase in the urinary elimination of Mn. This fact may also be explained by an augmented degradation of proteins rich in Mn as a consequence of exercise. This explanation can be reinforced by the role of Mn as an antagonist of iron (Fe) [47], a critical element in aerobic metabolism. In this respect, the obtained results could be produced as a preventive body response to ensure optimal levels of Fe. Although the real cause of these changes is not entirely clear, the obtained result manifests a real influence of aerobic exercise on the body values of Mn.

The six months of the study did not produce significant changes of Mo in the serum or urine of the athletes.

In relation to Se, the lower serum concentrations of Se found among athletes after the aerobic training program could be explained by increased cellular metabolic demands in order to develop an adequate antioxidant response induced by the oxidative stress linked to aerobic exercise. Furthermore, considering that in addition to these results, a diminution was observed in the urinary elimination of this element without changes in the daily intake, it seems clear that this framework suggests a possible adaptive response of the body to retain this element in order to prevent major losses and to ensure adequate body concentrations of Se to meet exercise-induced demands.

A diminution was observed in the serum concentrations of V among the athletes after the training period that was accompanied by an increase in the intake of this element. This fact could seem contradictory, but these results could be explained by a chronic redistribution of this element from blood to bone, excretory tract or adipocytes, as has been previously reported [39, 48]. However, this fact is not directly linked to physical exercise so it seems most likely that the obtained results may be mainly due to an increased use of this element to exert its insulin mimetic function either to maintain homeostasis or to enhance the metabolism of carbohydrates or recovery after exercise.

Regarding the Zn results, the significant increase found in the serum among the athletes at the end of the study could be due to an increased disposal from muscle reserves, as it is known that the greater proportion of body Zn is found in skeletal muscle (50–60%) and bone (25–30%) [49].

These increased serum values of Zn could be explained by different hypotheses. The first one could be based on the anti-inflammatory role of Zn, with the increase in serum being a possible adaptive mechanism used by the athletes to protect their body against inflammation resulting from strenuous physical activity. The second one, could be based on the antioxidant role of this mineral. As aerobic athletes are exposed to increased oxidative stress this result could be explained as a response to prevent oxidative damage and to reduce muscle fatigue.

In all cases, it seems clear that physical exercise affects the serum values of this element, so, it could be assumed that a functional Zn redistribution may occur between tissues during exercise in order to meet the demands induced by physical training, affecting the serum values. For the abovementioned reports, the obtained results could have a positive impact among the athletes due to a major bioavailability of this mineral, a critical fact for physical performance, and could be explained as an adaptive response to overcome the physical demands of training.

## Conclusions

It can be concluded that, except for V, all the mineral studied presented different serum or urinary concentration in athletes with respect to sedentary people in basal conditions, before and after de study. This could be related to mechanisms of adaptation to high intensity aerobic training.

Six months of aerobic training among well-trained athletes can induce important changes in serum and urine concentrations of several essential elements. The main findings in this survey were an increase in the serum concentrations of Zn and a decrease in the concentrations of serum Mn, Se and V that can alter the athlete’s physical capacity. The athlete’s body can develop changes in urinary elimination of some elements by reducing (Cu and Se) or increasing (Co, Mn, and Zn) excretion rates to maintain the organism in a good state and prevent negative effects.

The results obtained seem to manifest a possible body need of several elements, like Mn, Se and V, a fact which may indicate specific nutritional supplementation requirements, due to reductions in urinary elimination, to maintain concentration as this situation could lead to risks for the performance of the athletes if it is maintained for long periods. In all cases, further research is required to discover, in more detail, the specific causes of these changes and the possible consequences.

## Data Availability

All data generated or analyzed during this study are included in this published article.

## Abbreviations

AG: Athletes group
CG: Control group
Co: Cobalt
Cu: Copper
DNA: Deoxyribonucleic acid
GPx: Glutathione peroxidase
H2O2: Hydrogen peroxide
ICP-MS: Inductively coupled plasma mass spectrometry
LDH: Lactate dehydrogenase
Mn: Manganese
Mo: Molybdenum
Se: Selenium
SOD: Superoxide dismutase
V: Vanadium
Zn: Zinc
Σ4: Sum of 4 skinfolds
Σ6: Sum of 6 skinfolds
